# Intraspecific Niche Evolution in a Drought Deciduous Shrub With Implications for Climate Resiliency

**DOI:** 10.1002/ece3.72816

**Published:** 2026-01-07

**Authors:** Katie J. Pennartz, Evan P. Tanner, Megan K. Clayton

**Affiliations:** ^1^ Caesar Kleberg Wildlife Research Institute Texas A&M University‐Kingsville Kingsville Texas USA; ^2^ Texas A&M AgriLife Extension Service Uvalde Texas USA

## Abstract

Whitebrush ( *Aloysia gratissima* ) is a drought deciduous shrub species with two geographically distinct populations occurring in North and South America where they experience different climatic conditions. This scenario of isolated populations existing under different climatic conditions provides an opportunity to explore the use of species distribution models (SDMs) to identify and classify climatic niche evolution. Our goal was to identify intraspecific patterns of niche evolution by exploring the variation in environmental variables that underlie and constrain distributions and then apply this information to provide insight on variation in species response to changing conditions at the population level. We conducted a principal component analysis to compare climatic conditions present throughout each population in addition to identifying geographical areas characterized by climate novelty. We built SDMs with MaxEnt to characterize the climatic niche at species and population levels. Reciprocal spatial transfers between population models were conducted to evaluate niche equivalency and categorize evolutionary trends as conservative or divergent. Lastly, we projected models onto future climatic conditions to evaluate the consequences of niche evolution on climate resiliency at the population level. Comparison of abiotic conditions between populations indicated evidence of non‐analogous climates and low niche overlap in both environmental and geographic spaces, suggesting that niche divergence occurs between populations. The differences in realized climatic niche of the populations are likely a result of exposure to novel environmental conditions within the dispersal vicariant population. Comparison of climate conditions and identifying the driving evolutionary forces shaping a species or population's niche will allow for adaptive management strategies in current and future climate scenarios by accounting for the spatial and temporal variation of species' responses to these pressures.

## Graphical abstract

The abstract explains that *Aloysia gratissima* (whitebrush) has two geographically isolated populations in North and South America that experience distinct climatic conditions. Using species distribution models, principal component analysis, and reciprocal spatial transfers, the study tested for evidence of climatic niche divergence versus conservatism between populations and projected future suitability under climate change. The findings show low niche overlap and evidence of divergence driven by exposure to novel climates, which highlights how understanding these evolutionary patterns can guide adaptive management of species' responses to changing environmental conditions.

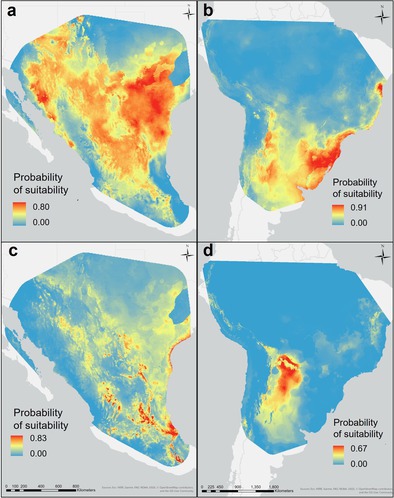

## 1 Introduction

Recent decades of climatic change, characterized by increased frequency of extreme temperature events and shifts in precipitation regimes, have been facilitated by anthropogenic activity that have increased extinction risk and selection pressure on terrestrial vegetation through exposure to novel environmental conditions within their established ranges (Parmesan 2006; Pillet et al. 2022). Since an estimated 98% of vegetation species distribution limits are defined by a reduction in climate suitability, these environmental changes will continue to result in habitat loss and distribution shifts for an increasing number of species (Zimmermann et al. 2009). Despite the limited individual mobility of flora, these species are not without coping strategies. Ecological and evolutionary processes have facilitated population persistence over the course of millions of years, far longer than the current climatic conditions have existed (Evans et al. 2009; Holt 2009). Through evolution of both their realized and fundamental niches, terrestrial vegetation species have kept pace with dynamic abiotic conditions by way of range shifts and climatic tolerance adaptations (Ackerly 2003; Holt et al. 2022).

Evolutionary niche theory serves to categorize possible species' responses to novel conditions as conservative or divergent processes based on niche dynamics. Low variability across space and time of the realized niche demonstrates a conservative pattern wherein evolution of the fundamental niche is constrained to mimic its ancestral role (Wiens et al. 2010). Conversely, evidence of altered relationships between abiotic conditions and environmental suitability indicates divergence, a deviation from the ancestral niche (Ackerly 2003; Wiens et al. 2010). Whether exhibiting conservatism or divergent behavior, niche evolution is the result of dynamic processes interacting at multiple scales that shape species' development (Holt 2009).

The direction and rate of niche shifts is contingent on a suite of intrinsic and extrinsic factors that invoke species‐specific responses, but spatio‐temporal climate variation is widely cited as the primary driver of niche dynamics (Ackerly 2003; Parmesan 2006; Slatyer et al. 2013). Distributions spanning large geographical areas are inherently composed of a larger range of environmental conditions indicating a wider tolerance or niche breadth (Slatyer et al. 2013).

Individuals existing on the edges of their range are generally considered to be experiencing conditions closer to their physiological limits and are therefore under higher selection pressure (Soberón and Peterson 2021). Alternatively, environmental conditions have been proposed to form a mosaic of stress resulting in non‐uniform selection pressure across the distribution (Soberón 2007). While species evolution is primarily constrained at range boundaries, adaptations that promote niche evolution are accumulated throughout the geographic extent (Soberón and Peterson 2021). As a result, the degree of climate heterogeneity experienced by a species influences how its niche evolves (Franks et al. 2014; Parmesan 2006). Adaptation of smaller evolutionary units, individuals or populations, to local conditions impacts the genetic and phenotypic diversity of that area (Eriksson and Rafajlović 2022; Franks et al. 2014). Species exhibiting high plasticity may have multiple co‐occurring phenotypes or a single dominant type determined by biogeographic location (Eriksson and Rafajlović 2022). Functional traits such as frost tolerance or drought resistance are not likely to vary within metapopulations or recently isolated populations due to the rate of trait evolution and few studies evaluate niche evolution over a phylogenetically meaningful timeline (Pearson 2010). Even so, the subtle shifts caused by local adaptations can lead to populations of the same species filling disparate niche space given time and opportunity. Their independent evolution is perpetuated when limited dispersal capability or biogeographical barriers (i.e., vicariance) block gene flow between populations (Wiens and Graham 2005).

Natural selection is inherently a conservative process, promoting adaptations that increase fitness within the current niche (Muscarella et al. 2014; Fick and Hijmans 2017). Range shifts have been increasingly documented as populations conserve their fundamental niche by altering their realized niche. Global trends show contraction at warm edges paired with expansion at cool edges as species track their environmental niche up elevation gradients and latitudes (Parmesan and Singer 2022). Even so, species are exposed to novel conditions as they disperse into novel biogeographical territory. Differences in topography and novel combinations of conditions will require evolution of the fundamental niche or bar the establishment of successful populations in the newly accessed areas (Moore and Donoghue 2007). Globally, there has been an average rise in temperature of 1°C since the beginning of the 20th century (Willi and Van Bskirk 2022). The theoretical rate of climatic niche evolution accommodates a change of 1°C per million years, and even rapid trait evolution events are estimated to require 10,000–100,000 years (Evans et al. 2009). Thus, the persistence of our current vegetation communities is unclear given these novel anthropogenic press dynamics. Understanding the nature of niche evolution is fundamental for implementing effective conservation strategies against the global decline in biodiversity that has led to loss of ecosystem services (Razgour et al. 2019). The ecological and evolutionary processes driving evolution interact at multiple spatial and temporal scales, requiring careful consideration of tools used to model niche behavior (DeMarche et al. 2019).

Empirical evidence of niche evolution and its consequences have been researched for use in developing theory, implementing conservation practices, and predicting future challenges (Elith and Leathwick 2009). Species distribution models (SDMs) are a common tool for describing the multidimensional environment a species occupies through linking geographical occurrence data to spatially explicit environmental data (Elith and Leathwick 2009). Comparing model predictions between populations or closely related species can identify changes in distribution drivers and climatic tolerances that provide insight on how a niche has evolved (Broennimann et al. 2012). The species' habitat requirements inferred by the model are able to be extrapolated to predict where suitable areas occur across space and time (Zurell et al. 2012). Current available methods operate on the assumption of niche conservatism and are unable to predict how variation in adaptive capability within species will affect range dynamics (DeMarche et al. 2019). Developing protocols for identifying niche divergence and evaluating its effect on performance of transference models is a critical step in forming the accurate predictions necessary to implement conservation strategies (Qiao et al. 2019).

Our goal was to identify empirical evidence of intraspecies niche evolution to provide insight on the causes and consequences of evolutionary patterns through analysis of correlative distribution models. We selected whitebrush ( *Aloysia gratissima* ), a drought deciduous shrub belonging to a genus that originated and diversified in South American approximately 40 million years ago (mya) before dispersing to North America approximately 20 mya (Olmstead 2013). Whitebrush typically grows 0.9–3 m tall and may occur as scattered individuals or in dense thickets. It has a multi‐stemmed woody growth form and produces small, narrow leaves along with white to blue flowers from March through November (SEINet – AZ/NM Node 2022). These traits, combined with its ability to defoliate during periods of water stress, allow the species to persist in climates characterized by high variability. The current distribution of whitebrush consists of two isolated metapopulations, hereafter referred to as the Northern and Southern populations based on continental location. The disjunct distribution pattern is an example of niche conservatism operating at a global scale in conjunction with climatic patterns creating ecologically comparable habitats at similar latitude (Wen and Ickert‐Bond 2009). After establishment and then later loss of genetic connectivity, the populations' niches evolved in isolation as natural selection acted to maximize fitness in the new and ancestral environments independently (Sexton et al. 2014). The variation in environmental conditions experienced by these populations (i.e., colder growing seasons and shorter frost‐free periods in the North American population versus hotter, drier conditions and more pronounced seasonal drought in the South American population) created distinct selective pressures, and, in the absence of genetic exchange, provided ample opportunity for independent local adaptation and niche divergence (Ackerly 2003). Plants living near the edge of their physiological limits are already constrained by environmental stress, which reduces their capacity to tolerate further changes in temperature or moisture availability (Parmesan and Singer 2022). We built upon previously proposed frameworks for evaluating climatic niche equivalency using correlative SDMs and quantitative multivariate climate analysis to identify empirical evidence of the patterns and consequences of niche evolution. Our objectives were to (1) assess patterns of niche evolution through comparing the primary climatic drivers structuring the ecological niches within isolated populations and (2) assess the impacts of differing climate conditions on population level climate relationships both now and in the future. Specifically, we asked: (1) Do isolated populations exhibit evidence of niche divergence, as indicated by limited transferability of ecological models across regions? and (2) When models are not transferable, can this lack of transferability be explained by underlying climate dissimilarity between the two regions? We believe that evidence of niche divergence facilitated by genetic isolation and exposure to disparate climate conditions would appear as a lack of transferability between population level ecological models. In the opposing scenario, conservative evolution would maintain similar functional niches between populations resulting in some degree of transferability given analogous climate conditions. By projecting our models built under current climate conditions into future scenarios we gain insight on how patterns of niche evolution will affect climate resiliency at a population level. We believe the failure to adapt to novel conditions exhibited by conservative evolutionary lineages could be a disadvantage depending on local trajectories of climate change that results in range contraction.

## 2 Materials and Methods

### 2.1 Study Area

The distribution of whitebrush extends from approximately 40 degrees north of the equator to 35 degrees south at elevations between 25 and 2500 m (GBIF, ). Populations occupy arid and semi‐arid environments within temperate and subtropical climate zones; therefore, presence is not continuous throughout the species' range (SEINet – AZ/NM Node 2022; Wiens and Graham 2005). This pattern of amphitropical disjunction, where a species is distributed at comparable latitudes on opposite sides of the equator without occurring in the tropics, present in the species' distribution will result in three focus study areas: the full extent of the species' distribution including the vast unoccupied region between populations, an area defined by the northern population observations in North and Central America, and an area defined by the southern population observations in South America. The southwestern United States and North and Central Mexico host the northern metapopulation, while the southern metapopulation is found in Argentina, Bolivia, Brazil, Paraguay, and Uruguay. Past research has found similar climatic signals between the two broad geographical areas encompassing the population distributions (Paruelo et al. 1995).

### 2.2 Species Distribution Models

SDMs were built using the MaxEnt v 3.4.4 software, which operates under a presence/pseudo‐background framework to estimate probability of suitability based on georeferenced occurrence data and spatially explicit predictor variables (Phillips and Dudík 2008). Observation records were collated from the Global Biodiversity Information Facility (GBIF), Southwest Ecological Information Network (SEINet – AZ/NM Node 2022), and Kew Herbarium online data portals to create a presence only dataset spanning from 1960 to 2018 (GBIF 2022; SEINet – AZ/NM Node 2022). Occurrences were divided by continent into northern (*n* = 196) and southern (*n* = 140) populations (Table S1) Details on the steps taken to rarify occurrence data and determine the extent of our study area can be found in the Appendix S1 (Zurell et al. 2020).

Monthly averages of historical (1960–2018) and future (2041–2100) climate data were downloaded from the WorldClim repository and converted into 19 bioclimatic variables using the “dismo” package in R v 4.1.2 (Fick and Hijmans 2017). In an effort to include possible species‐specific distribution constraints, an additional predictor variable was calculated to represent temperature extremes during the coldest quarter (Zimmermann et al. 2009). Variables representing future climate conditions were calculated from an ensemble mean of eight global climate models representing the socioeconomic scenario pathway 5–8.5 (SSP5−8.5; Fick and Hijmans 2017; Mahony et al. 2022). We created models under the SSP5–8.5 pathway, a very high‐ greenhouse gas emissions scenario in which CO_2_ emissions are tripled by 2075 and result in ~8.5 W/m^2^ radiative forcing by 2100, as it closely reflects current global emission trajectories. We chose to focus on climate variables that influence the fundamental niche in an effort to produce output that would reflect the potential environmental niche of the species (Elith and Leathwick 2009; Zurell et al. 2012). After removal of highly correlated variables, 10 candidate variables were considered for model inclusion (Table S2).

We adapted the SDM transferability criteria presented by Randin et al. 2006 to evaluate the pattern of intraspecies niche evolution through the inclusion of comparisons in environmental space. The original framework assesses model transferability through a geographic lens with the following criteria as applied to our study: (1) baseline models must show comparable performance among themselves, (2) baseline models must retain comparable predictive power when spatially transferred, and (3) the spatially explicit probability of suitability predictions must match between the baseline and spatial transfer models within each study area (Randin et al. 2006). Assuming conservatism or niche equivalency as the null hypothesis, our additional criteria required that (4) climate relationships are maintained between populations and (5) comparison of population niche in environmental space is non‐significant (Loera et al. 2012; Nakazato et al. 2010).

### 2.3 Baseline Estimation

Three SDMs were built to represent the climatic niche at the species and population levels, each consisting of 30 replicates which were evaluated using a bootstrapping technique with an 80:20 training:testing occurrence data ratio (Elith et al. 2011). Further information on settings and technical details for all presented models can be found in the ODMAP reports, which are a standardized way of documenting SDM development, under Appendix S1 (Fitzpatrick et al. 2021; Zurell et al. 2020). We initially built a species‐ wide model to select the predictor variables that would be used in all following SDMs to allow for direct comparison and avoid biasing outcomes toward one population over the other (Elith et al. 2011). We used geographical subsets of our environmental and occurrence data to model the populations independently, with regularization values and feature class settings selected through the “ENMeval” package in R (Tables S3 and S4) (Muscarella et al. 2014; Warren and Seifert 2011). The population‐climate relationships characterized in these models will serve as the basis for the following spatial and temporal transfers.

Analysis was performed in R v 4.1.2 and ArcGIS v 10.8.1 (ESRI 2023; R Core Team 2022). Model performance was evaluated using the AUC metric, which accounts for sensitivity and specificity to evaluate model accuracy on a scale of 0–1 (Fielding and Bell 1997). Scores below 0.5 are indicative of models with less predictive power than random chance and scores above 0.7 are considered to represent an acceptable level of discernment in SMDs (Allouche et al. 2006; Fielding and Bell 1997). Our threshold dependent metric was evaluated by converting the median logistic probability of suitability predictions to binary form using the maximum test sensitivity plus specificity threshold rule (Table S5) (Liu et al. 2013). The standardized nature of this metric is due to the threshold rule being held constant across all model evaluations and allows for comparison of model performance (Liu et al. 2013; Merow et al. 2013).

We compared the distributions of the marginal response curves generated by the MaxEnt algorithm for each predictor variable between populations using the Kolmogrov–Smirnov two sample test (Berger and Zhou 2014). This allowed us to assess differentiation in population‐ climate relationship trends for each variable individually and provided insight on which climatic conditions were driving the evolutionary process (Parmesan 2006).

### 2.4 Model Transference

#### 2.4.1 ExDet: Extrapolation Detection

We first assessed the transferability of our population models by identifying areas of novelty or dissimilar climatic conditions between populations that would introduce extrapolation, where the model makes predictions outside the conditions it was trained under, into the modeling process (Qiao et al. 2019; Yates et al. 2018). Projecting correlative SDMs onto areas that exist outside of the range of values the model was trained to can result in poor predictive power and mischaracterization of species‐environment relationships (Randin et al. 2006). We chose the ExDet software to identify climatic novelty between populations as it provides spatially explicit reports on two types of novelty: univariate (type 1; pixels outside the univariate range of conditions within the reference region) and multivariate (type 2; pixels within univariate bounds, but representing novel multivariate combinations), while also identifying the variables contributing to measured dissimilarity (Mesgaran et al. 2014). Dissimilarity values are calculated based on the Mahalanobis distance metric and range from [−∞,0] for type 1 novelty and [0, +∞] for type 2 novelty (Mesgaran et al. 2014). Pixels assigned a value of zero indicate analogous conditions between the reference and projection areas where no extrapolation will be necessary by the transferred models. Decreasing negative values measuring type 1 novelty indicates increasing dissimilarity (Mesgaran et al. 2014). Type 2 novelty is present at values greater than 1, with larger values representing greater dissimilarity of multivariate conditions between the reference and projection areas (Mesgaran et al. 2014). By characterizing novel conditions between populations, we were able to characterize the likely drivers of disparate climate‐driven evolution for our species (Ackerly 2003; Wiens and Graham 2005).

After accounting for additional sources of model uncertainty by identifying dissimilar climate conditions between the two study areas, we spatially transferred our top performing baseline population models to evaluate the degree of equivalency of the estimated metapopulation niches using reciprocal models (Brown et al. 2017; Randin et al. 2006). More specifically, the observed environmental relationships between occurrences and climate conditions in the northern metapopulation were applied to predict geographical occurrences in the southern extent and vice versa. In the case of niche conservatism where the physiological requirements that inform the fundamental niche have remained largely unaltered over time, we would expect models to perform better than random given analog conditions between the population's representative climate variables such as minimum temperature requirements for growth, tolerance to drought stress, and other physiological limits on resource acquisition (Glor and Warren 2011; Warren et al. 2008).

We estimated the geographic niche overlap of the baseline metapopulation models with their transfer counterparts to evaluate climatic niche equivalency using “ENMtools” in Program R v 4.2.1 (Warren et al. 2010). We calculated Schoener's D scores to estimate niche equivalency, which have possible values ranging from 0 (no overlap) to 1 (complete overlap) (Schoener 1974). We would expect values closer to 0 to be associated with divergence and those near the maximum to indicate conservatism. We ran a comparative hypothesis test using the identity.test function in the “ENMtools” package to measure niche similarity using Schoner's D metric between the niches estimated by the baseline population models and their spatial transfer counterparts (I.e. compared the baseline model of the northern population to the spatially transferred southern population model) (Warren et al. 2010). When the similarity score of the input models is significantly lower than that of the pseudo‐replicates, the null hypothesis that the population niches share an identity is rejected in favor of evidence of niche divergence (Warren et al. 2010).

To complement the spatially explicit similarity measures calculated using ExDet, we also conducted a principal component analysis (PCA) based on the climatic conditions present at occurrence locations to compare the population's niches in environmental space using the R package “prcomp” (Nakazato et al. 2010). Occurrences were grouped based on geographic location and a permutational multivariate analysis of variance (PERMANOVA) was used to assess the statistical difference between apparent preferred habitat in each population (Nakazato et al. 2010; Slatyer et al. 2013). A PERMANOVA is a semiparametric method of determining significance based on dissimilarity measures that does not assume multivariate normality (Anderson 2017). We calculated the dissimilarity matrix using the Mahanalobis distance metric and ran the analysis with 999 permutations (Anderson 2017; Mesgaran et al. 2014). For our final round of models, the baselines were transferred again, this time temporally, by projecting climatic suitability relationships onto simulated climate variables representing future conditions within their historic geographical range. Three models were built per population, each projected onto an ensemble mean of eight global climate models (Table S6) from the Coupled Model Intercomparison Project Phase 6 (CMIP6) release covering a 20‐year span (2041–2060, 2061–2080, 2081–2100) (Fick and Hijmans 2017). The global climate models were representative of possible conditions under the shared socioeconomic pathway 585, which depicts a future with strong economic growth through fossil fuel development (Gidden et al. 2019). Mapping the predicted changes in available habitat allowed us to observe the temporal effect of altered environmental conditions on geographic niche boundaries (Elith and Leathwick 2009; Randin et al. 2006).

The median logistic suitability values (grain = 1.1 km^2^) for each time period model were overlaid in ArcMap v 10.8.1 to calculate the predicted change in environmental suitability under future climatic conditions (Pearson 2010). Areas were categorized based on changes in binary suitability predictions across our temporal transfer models. Predictions were adapted from logistic probability values using the maximum test sensitivity plus specificity threshold rule (Table S5) (Liu et al. 2013). Designations were based on persistence of suitability and the possible categories were unsuitable, gain of suitability, loss of suitability, and stable suitability.

## 3 Results

### 3.1 Baseline Results

Our final northern and southern population distribution models performed well with AUC scores of 0.81 and 0.92, respectively, and exhibited acceptable standardized test omission (STO) error rates of 0.24 (SD = 0.12) and 0.2 (SD = 0.08). Although both population models were informed by the same set of predictors consisting of four temperature and three precipitation related variables (Table 1), there was disparate contributions to model accuracy gain across populations (Figure S1). For the northern population, distribution appeared to be constrained by both moisture and thermal conditions with mean temperature of the driest quarter (44.3%), precipitation of the wettest month (17.2%), extreme temperature events of the coldest quarter (13.2%), and precipitation of the wettest quarter (12.4) comprising our top four informers. In contrast, the southern model derived the majority (83.1%) of its predictive power from temperature related variables, primarily isothermality (62.4%). Mean temperature of the driest quarter (9.7%), precipitation of the driest month (9.3%) and diurnal range (8.3%) followed next in the rankings with lower contributions (Table 1).

**TABLE 1:** The range, the value associated with peak suitability based on marginal response curves, and the percent contribution to gains in model accuracy for climate variables included in the final MaxEnt base population models of whitebrush ( *Aloysia gratissima* ).

	Northern			Southern		
Bioclimatic variable	Range	Peak suitability	Percent contribution (%)	Range	Peak suitability	Percent contribution (%)
0	0.5–4.0	2.8 (SD)	13.2	0.2–2.4 (std dev)	1.9 (std dev)	2.7
2	4.7°C–23.4°C	5°C	2.6	4.5°C–20.2°C	24°C	8.3
3	24.8°C–80.7°C	42°C	6.3	34.6°C–97.8°C	48°C	62.4
9	−8°C to 33.4°C	15°C	44.3	−9.2 to 32°C	15°C	9.7
13	0–922 mm	0 mm	17.2	0–770 mm	120 mm	4.3
14	0–122 mm	42 mm	4.0	0–300 mm	90 mm	9.3
18	0–1290 mm	145 mm	12.4	0–1643 mm	1500 mm	3.2

*Note:* Climate variable IDs refer to 0—Extreme minimum temperature events of the coldest quarter, 2—mean diurnal range, 3—isothermality, 9 mean temperature of the quarter, 13—mean precipitation of the warmest quarter, 14‐ mean precipitation of the driest quarter, 18—mean precipitation of the wettest quarter (Fick and Hijmans 2017; Zimmermann et al. 2009).

**FIGURE 1:**
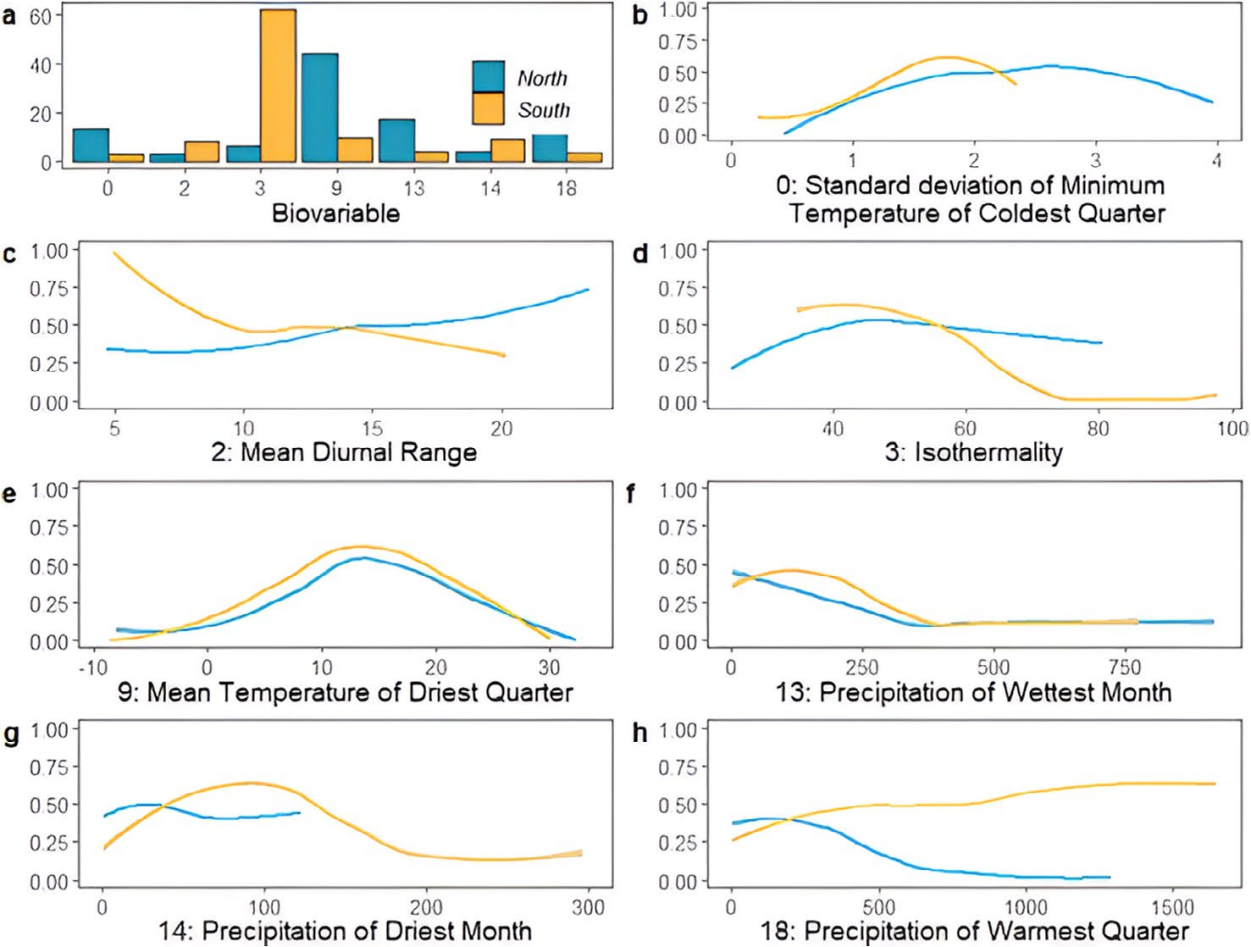
Selected biovariable contribution to baseline model performance (a) for the Northern (blue) and Southern (yellow) populations of whitebrush ( *Aloysia gratissima* ) accompanied by the probability of suitability in relation to the individual variables (b) extreme temperature events of the coldest quarter (standard deviation of mean temperature), (c) mean diurnal range (°C), (d) Isothermality (%), (e) mean temperature of the driest quarter (°C), (f) precipitation of the wettest month (mm), (g) precipitation of the driest month (°C), and (h) precipitation of the warmest quarter (mm).

Analysis of the marginal response curves indicated a statistical difference in the populations' climate relationships which failed to meet our fourth requirement (Table S7). Response curves representing mean temperature of the driest quarter and precipitation of the wettest month exhibited similar trends over comparable climatic ranges between populations (Figure 1). For the extreme temperature events of the coldest quarter variables, precipitation of the driest month, and isothermality, the general trend of the response was similar but differed in population exposure to environmental extremes (Figure 1). Lastly, the population models predicted disparate responses to mean diurnal range and precipitation of the warmest quarter variables. Probability of suitability in the northern population increases along with the temperature difference between day and night (mean diurnal range), while the opposite is true for the southern population (Figure 1). When considering the effects of precipitation in the warmest quarter, suitability declines with additional precipitation after 145 mm in the northern model but steadily increases in the southern model until peaking at 1500 mm (Figure 1a).

### 3.2 Transference Results

**FIGURE 2:**
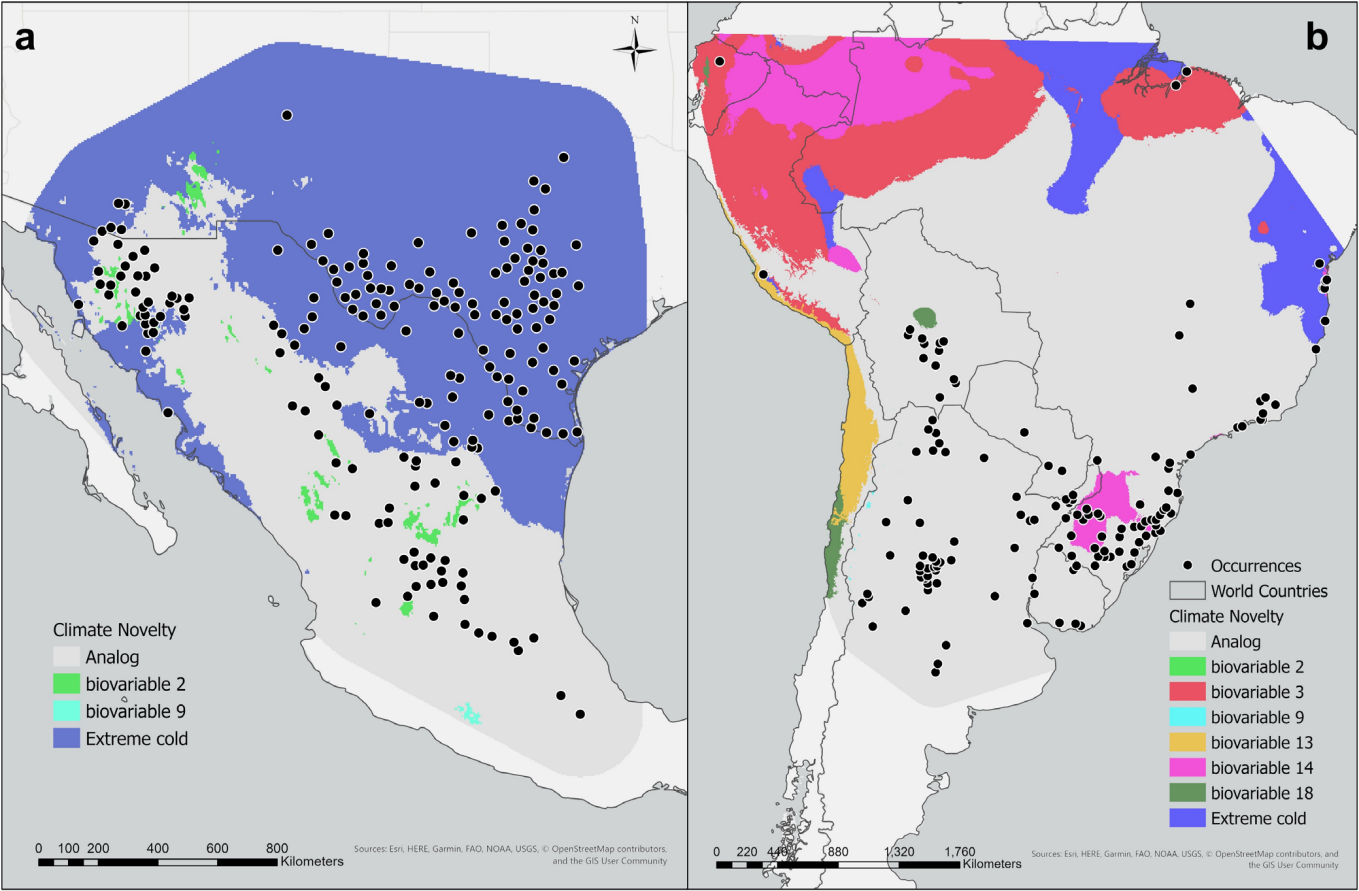
Geographical representation of novel univariate conditions present in the (a) northern and (b) southern populations of whitebrush ( *Aloysia gratissima* ) as determined by the ExDet software for predictor variables included in the final models (Mesgaran et al. 2014).

We detected novel areas representing climate dissimilarities within both population ranges. There was no evidence of dissimilarity arising from unique combinations of climate conditions and only type 1 or univariate causes were identified. Within the northern range, 64% of our occurrences were located in areas that experience extreme cold temperatures not present in the south, particularly on the leading edge of the distribution (Figure 2). Limited areas of novelty arising from mean diurnal range and mean temperature of the driest quarter were present within the range as well, but a lack of occurrences in these areas limited their impact on our objectives. Climate conditions unique to the southern extent occurred primarily along the coasts of South America and the trailing edge of the distribution encompassing 16% of our occurrences (Figure 2). All seven of our variables were represented in the non‐analogous areas, however only three (isothermality, precipitation of the driest month and extreme cold) were associated with our occurrence locations. In addition to a higher proportion of the northern population existing in non‐analog conditions, the degree of dissimilarity was stronger (−0.32) than for the southern occurrences (−0.09) (Figure S2).

#### 3.2.1 Spatial Transferability

**FIGURE 3:**
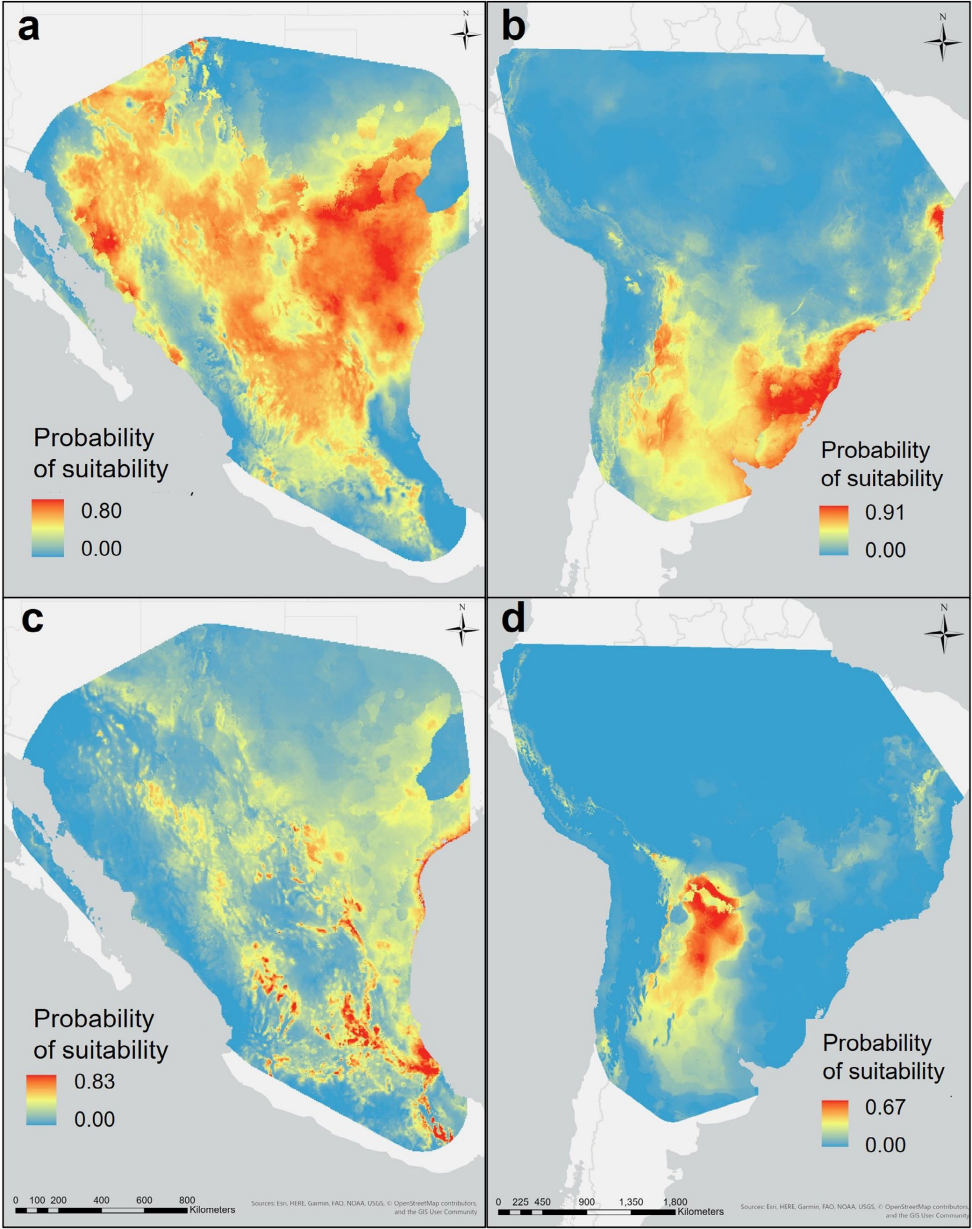
Logistic probability of suitability maps generated from the baseline (a, b) and spatial transference (c, d) models for whitebrush ( *Aloysia gratissima* ) populations in North America (a, c) and South America (b, d).

When we spatially transferred our population models, their predictive performance decreased drastically, failing to meet the second requirement, with the northern model omitting 84.18% of occurrences in the southern extent and the southern model categorizing 96.43% of northern occurrences as being in unsuitable areas (Figure 3). Considering these results, the measure of geographic niche overlap between the baseline prediction and spatial projection was higher than expected, with an estimated Schoener's D value of 0.696 in the northern study area and 0.475 in the southern study area. However, this still failed to meet the criteria necessary to assume niche conservatism (Warren et al. 2008). Moreover, the null hypothesis of shared niche identity was rejected (*p* < 0.01, α = 0.05), further providing empirical evidence of niche divergence (Warren et al. 2008).

Comparison of the populations' climatic niches in environmental space (through our PCA) continued to highlight differences in the conditions characterizing their occurrence locations. Occurrence locations were mapped along the primary and secondary axis accounting for 52.2% and 21.7% of variation present in the dataset, respectively (Figure S3; Table S8). A PERMANOVA analysis indicated significant differences in the climate conditions characterizing occurrence locations between populations (*p* < 0.001, α = 0.05).

**FIGURE 4:**
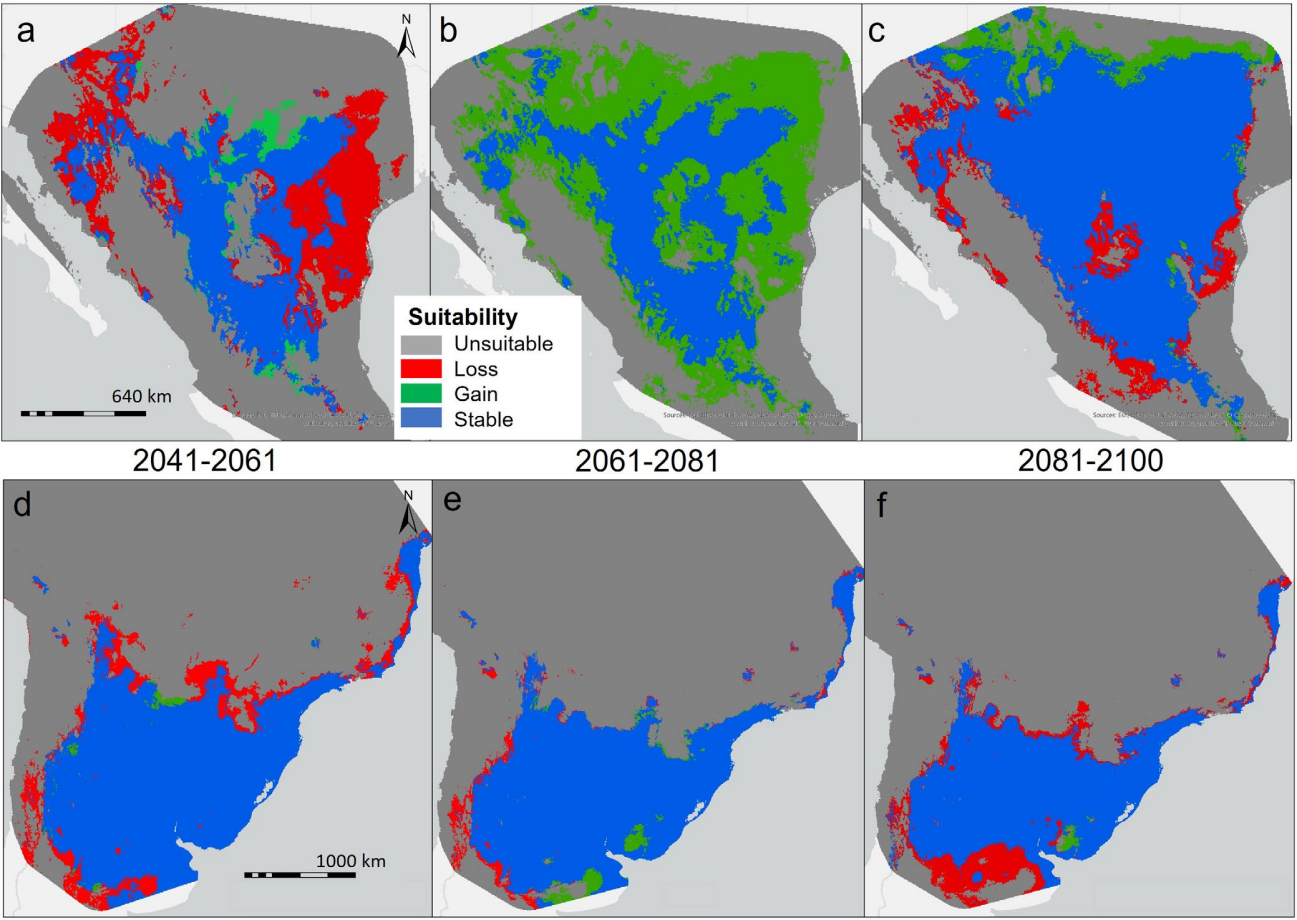
Categorical suitability maps for whitebrush ( *Aloysia gratissima* ) indicating changes in predicted suitability from current conditions to conditions representative of shared socioeconomic pathway 585 from the ensemble global climate model for the northern population in 2041–2060 (a), 2061–2080 (b), 2081–2100 (c), and for the southern population in 2041–2060 (d), 2061–2080 (e), 2081–2100 (f).

Our final suite of models that were temporally transferred to evaluate changes in predicted suitable area over time indicated the two populations will face disparate responses in future decades (Table 2). Under simulated future climate scenarios in North America, the majority of the range is expected to either remain suitable (32.46%) or become so (23.14%) by 2100, outpacing the predicted 5.99% loss of habitat. The gains in environmentally suitable areas will predominantly advance along the leading edge of the range northward, with more minor advances occurring at the trailing edge and along current longitudinal boundaries (Figure 4). While the expected percent loss in the southern range is comparable to its northern counterpart at 7.16%, it's predicted to affect both the trailing and leading edge of the distribution with minimal gains in area (0.16%) to offset the reduction in suitable area.

**TABLE 2:** Estimated change in suitable area for whitebrush ( *Aloysia gratissima* ) as estimated from MaxEnt and based on binary predictions adapted from logistic probability predictions using the maximum test sensitivity plus specificity threshold rule.

Classification	Northern prediction		Southern prediction	
Unsuitable: Never suitable	87.86 km^2^	38.41%	861.51 km^2^	75.94%
Loss: Currently suitable, becomes unsuitable and remains so	13.70 km^2^	5.99%	81.25 km^2^	7.16%
Gain: Currently unsuitable, becomes suitable and remains so	52.94 km^2^	23.14%	1.80 km^2^	0.16%
Stable: Always suitable	74.27 km^2^	32.46%	189.95 km^2^	16.74%

*Note:* Area measurements are presented as percent of study extent in addition to square kilometers due to the discrepancy in study area extent. Classification was based on long‐term suitability estimates for areas within the northern and southern population distributions as determined by our temporal transfer models informed by an ensemble mean of eight global climate models (Fick and Hijmans 2017; Mahony et al. 2022).

## 4 Discussion

By building upon previously presented frameworks for evaluating transferability of SDMs and identifying evolutionary patterns with ecological niche models, we were able to identify empirical evidence of intraspecies niche divergence between disjunct whitebrush ( *Aloysia gratissima* ) populations (Nakazato et al. 2010; Randin et al. 2006). Incorporating niche comparisons in environmental space as well as geographic space allowed us to evaluate aspects of the fundamental niche more directly, in addition to the realized niche to distinguish between their dynamics (Pearman et al. 2008). Evaluation of the causes and consequences of different evolutionary trajectories on population climate resiliency showed that exposure to novel conditions near physiological tolerances stimulated diversification in the northern population and that conservation of the ancestral niche decreased the southern population's predicted suitability under future climate scenarios (Pearman et al. 2008; Zimmermann et al. 2009). Being able to identify and predict the magnitude, direction, and pattern of intraspecific niche evolution will be necessary to inform conservation or management strategies as these dynamics will ultimately drive species performance under changing conditions in the Anthropocene (Pearson 2010; Razgour et al. 2019). Such strategies may include identifying and prioritizing populations with high adaptive potential for conservation, protecting or restoring habitat corridors that facilitate dispersal to suitable climates, and, conversely, using niche evolution insights to guide the control or removal of populations where the species is not desired or behaves noxiously (Joyce et al. 2013).

A species' or population's adaptive capacity is largely influenced by the spatial and temporal climate heterogeneity present within its historical range (Ackerly 2003; Franks et al. 2014). Detection of model extrapolation introduced by non‐analog conditions in transfer scenarios is considered best practice when interpreting distribution predictions and evaluating model performance (Qiao et al. 2019; Yates et al. 2018). In our study, it served to identify possible drivers of evolution by distinguishing differences in the primary constraining variables between disjunct distributions and provide insight on population specific behavior under altered climate conditions (Broennimann et al. 2012; Razgour et al. 2019). The initial dispersal event from South America is believed to have established a population in the climate analog of northern Mexico, following a conservative pattern of niche evolution on a global scale (Olmstead 2013; Wiens et al. 2010). As the new population expanded and was exposed to novel conditions, survival required adaptation in plant–climate relationships. Overcoming vicariance through avian facilitated long‐distance dispersal (i.e., endozoochory) events led to evolution of fundamental aspects that determine a species range in succeeding generations (DeMarche et al. 2019; Razgour et al. 2019). The increased exposure to cold temperatures at the leading edge of the northern distribution presented the opportunity for this species to begin to develop a cold tolerance, even though that trait was previously absent in this genus (Moroni et al. 2016). In this context, “cold tolerance” could involve traits such as increased frost hardiness through accumulation of protective solutes, altered phenology that reduces exposure of sensitive tissues to freezing events, or structural adaptations like thicker bark that insulates buds and vascular tissues (Moroni et al. 2016). As a result, whitebrush is one of three species in the Verbenaceae family that's range does not hold to latitude parallels (Olmstead 2013).

Results from the identity test indicating that our populations do not fill identical spaces in their systems combined with the significant differences when comparing similarity of single variable response curves provided insight on the direction and type of differentiation occurring. Comparisons of our probability of suitability response curves indicate that relationships to precipitation have been mostly conserved between populations (Figure 1e–g) with the exception of precipitation in the warmest quarter (Figure 1h). The disparate response observed with this variable and mean diurnal range of temperature (°C) (Figure 1c) occurs over a comparable range of conditions which suggests that variation in biotic influences are impacting the abiotic relationships (Liang et al. 2018). Identifying the primary constraints on geographic ranges from top performing model variables and possible exposure to novel conditions through extrapolation detection highlights the drivers and patterns of evolution that assists in interpreting the degree of differentiation (Palmquist et al. 2021). After observing the primary drivers and directions of adaptation, we must assess whether the changes that occurred will have significant effects on a species role within the system to accurately predict future behavior. Our spatially projected SDMs failed to meet the transferability criteria, showing poor performance even in areas characterized by analogous climates (Figure 3). These findings were taken as evidence that the degree of differentiation was significant enough to limit our ability to draw species‐wide inferences about plant‐climate interactions (DeMarche et al. 2019; Hällfors et al. 2016).

A pattern of asymmetric transferability has been commonly observed in SDM studies on vegetation species (Randin et al. 2006; Slatyer et al. 2013). While intraspecies adaptations in response to variation in the underlying environmental gradients offer a partial explanation, non‐ climatic factors contribute to niche divergence as well (Donoghue and Edwards 2014). Human impact, seed dispersal accessibility, and competition are factors that will vary between geographic locations and impact range dynamics (Donoghue and Edwards 2014). With the limited number of southern individuals present in areas of climate novelty compared with the northern population, asymmetric transferability could be expected. However, our results support an interpretation of niche contraction in the south coupled with a shift or expansion in the north. Through a PCA, we were able to compare the background climate conditions as well as those that characterized occurrence locations to investigate apparent habitat associations within the context of availability (Nakazato et al. 2010). While climate analogs exist between the study extents, individuals appear to be associated with differing conditions within their extents. Reductions in the range of suitable conditions are often preceded by a failure to adapt when encountering novel conditions causing loss of marginal habitats (Broennimann et al. 2012; Slatyer et al. 2013).

Rapid changes in climate have historically preceded mass extinction events and are currently contributing to local extinctions and the global declines in biodiversity (Nadeau et al. 2017). Populations with a demonstrated inability to adapt to new conditions will face higher selection pressures as they are forced to shift their geographical range to track suitable environmental conditions (Soberón and Peterson 2021). Adaptation of the realized niche or shifting provides a quicker response option than the millennia long process of developing new climatic tolerances, but populations may still be unable to keep pace with local trajectories of change (Burrows et al. 2011). The rate of range expansion is directly tied to species life history traits such as longevity and reproductive strategy and can be halted entirely by anthropogenic or geographic barriers to dispersal (Thomas et al. 2004). Anthropogenic barriers may include large‐scale agricultural conversion, urban development, and transportation infrastructure, which fragment habitat and block dispersal corridors (Thomas et al. 2004). In North America, for example, intensive agriculture and road networks create discontinuities in otherwise suitable areas, while in South America, expanding pasturelands and deforestation for cropland similarly disrupt dispersal pathways (Liang et al. 2018). As the gradual climate gradients that promote evolution are replaced by a mosaic of stress acting within distributions, the frequency of these failure events is expected to increase (Jump and Peñuelas 2005; Nadeau et al. 2017). While contraction of the fundamental niche does not always equate to restriction of the realized niche, our temporally transferred southern population model implies loss of suitable habitat along the geographical edges of the distribution (Slatyer et al. 2013; Soberón 2007). In contrast, we predicted vast gains in suitable area in the northern population around the margins and particularly in the leading edge of the distribution. This northward range expansion does not appear to be a possibility if the northern population was operating with the same level of adaptive capability as the southern population. This reduction in suitable habitat for a population with narrower niche breadth compared with its counterpart supports the idea that conservative tendencies can increase climate risk for a species or population by lowering resiliency, which depends on temporal and spatial genetic variation and adaptability (Hällfors et al. 2016; Razgour et al. 2019).

Beyond their theoretical importance, our findings have practical implications for the management of whitebrush in rangelands. The predicted contraction of suitable climate space in southern populations suggests that local declines may reduce the need for active control in some areas, while the potential for northern expansion highlights regions where encroachment pressure may intensify. Anticipating these dynamics could help land managers allocate resources more efficiently, focusing brush control where expansion is most likely (Joyce et al. 2013). In addition, recognizing that northern populations show evidence of greater adaptive capacity emphasizes the importance of monitoring these leading‐edge populations for early signs of spread. Integrating niche modeling with field‐based research on reproduction and response to treatments could support the development of more effective, region‐specific strategies for managing this species.

There have been a few studies using correlative prediction models to investigate patterns of adaptation and speciation over phylogenetically meaningful timelines (Franks et al. 2014).

SDMs are often implemented in conservation efforts although their static nature ignores the possible impacts of genetic or phenotypic variation present among individuals (Hällfors et al. 2016). The spatial scale of our study areas is larger than that of individual dispersal capacity, suggesting the possibility of genetic or phenotypic variation present at an even finer scale than the one we selected (Holt 2009). However, the purpose of our model was to assess the presence of niche differentiation within a widely distributed species rather than identify geographical areas for implementing management strategies. In a similar vein, the static assumption made by correlative SDMs was not a cause for concern when evaluating niche equivalency, although we may be ignoring niche dynamics in our temporal transfers as further local adaptation or phenotype development could occur on this timeline (Hällfors et al. 2016; Razgour et al. 2019). Adaptive management strategies for persistent conservation must be a reiterative process with intervals dependent on the local rate of change and species‐specific characteristics (Joyce et al. 2013).

Although these considerations frame the scope of our approach, additional limitations should also be acknowledged. Our modeling framework was restricted to climate variables, and thus our projections reflect only climate‐driven dynamics (Elith and Leathwick 2009). Non‐climatic drivers such as land use change, soil characteristics, disturbance history, and biotic interactions were not incorporated, yet may strongly influence the realized distribution of whitebrush by either amplifying or buffering the effects of climate (Liang et al. 2018). In addition, our evaluation of climate analogues relied on contemporary climate data, with the assumption that these differences have remained relatively consistent over the tens of thousands of years during which the species has evolved. While this approach is standard in correlative modeling, it simplifies the historical variability of climates. Moreover, although our results are framed in the context of niche evolution, evolutionary change often unfolds over millennia, whereas our projections address ecological responses to climate in the coming decades (Holt 2009). These interpretations should therefore be viewed as identifying potential ecological responses to shifting climates rather than forecasting rapid evolutionary adaptation.

Our results show the consequences of 20 million years of isolated evolution on population level adaptive capacity and its implications on model transferability, providing an example of the shortcomings of using poorly designed ecological models in management planning (Pearson 2010; Yates et al. 2018). To create distribution models beneficial to conservation efforts, appropriate scales will need to be selected to account for spatially and temporally nonhomogeneous genetic and phenotypic adaptations that shape ecosystem roles. Investigation of ecological niche evolution in species or clades that have exhibited a propensity for change through development of their genotype or phenotype will contribute valuable insight to our understanding of speciation and adaptation. Generating knowledge in this field will be necessary to mitigate species and community level consequences for terrestrial vegetation species experiencing rapidly changing climate conditions in future decades (Joyce et al. 2013; Palmquist et al. 2021).

## Author Contributions

**Katie J. Pennartz:** conceptualization (equal), data curation (lead), formal analysis (lead), investigation (equal), methodology (equal), writing – original draft (lead), writing – review and editing (equal). **Megan K. Clayton:** funding acquisition (equal), project administration (equal), supervision (equal), validation (equal), writing – original draft (supporting), writing – review and editing (equal). **Evan P. Tanner:** conceptualization (equal), data curation (supporting), formal analysis (equal), funding acquisition (equal), project administration (equal), resources (lead), supervision (equal), validation (equal), writing – original draft (supporting), writing – review and editing (equal).

## Conflicts of Interest

The authors declare no conflicts of interest.

## Supporting information


**Appendix S1:** ece372816‐sup‐0001‐AppendixS1.docx.

## Data Availability

All data required to reproduce this research is available at DOI: https://doi.org/10.5061/dryad.xpnvx0kqh. This is a peer review only link that will be updated at the time of publication.
