# Supplementary material for: Intraspecific Niche Evolution in a Drought Deciduous Shrub With Implications for Climate Resiliency
Source: Ecol Evol. 2026 Jan 7;16(1):e72816. doi: 10.1002/ece3.72816 (PMC12778428; doi:10.1002/ece3.72816)

**Supplementary materials**

**Table S1.** Possible whitebrush (*Aloysia gratissima*) sample sizes of population occurrences when rarified based on climate heterogeneity assuming different combinations of spatial resolution and number of climate categories calculated using the ‘SDMtoolbox’ in ArcMap v 10.8.2. Initial sample sizes were 564 occurrences in the northern population and (Brown et al., 2017; ESRI, 2023). Selected parameters and resulting sample size are denoted by the symbol *.

| Moving window size  (resolution) | Max climate categories | Northern  sample size | Southern  sample size |
| --- | --- | --- | --- |
| 5 | 3 | 195 | 162 |
| 5 | 5 | 195 | 138 |
| 10* | 3* | 196* | 140* |
| 10 | 5 | 199 | 141 |
| 20 | 3 | 191 | 141 |
| 20 | 5 | 296 | 142 |

**Table S2**. Variables considered for inclusion in the baseline Maxent models to estimate the climatic niche of whitebrush *(Aloysia gratissima)* and the average variable contribution to model accuracy gain. The symbol * denotes inclusion in the final model.

| ID | Description | Calculation |
| --- | --- | --- |
| 0* | Extreme minimum temperature events of the coldest quarter (°C) | Standard deviation of daily minimum temperatures |
| 2* | Diurnal range (°C) | Mean of monthly*(max temp - min temp.) |
| 3* | Isothermality (°C) | (BIO2/BIO7) (×100) |
| 8 | Mean temperature of wettest quarter |  |
| 9* | Mean temperature of driest quarter (°C) |  |
| 13* | Precipitation of wettest month (mm) |  |
| 14* | Precipitation of driest month (mm) |  |
| 15 | Precipitation seasonality | Coefficient of variation |
| 18* | Precipitation of warmest quarter (mm) |  |
| 19 | Precipitation of coldest quarter (mm) |  |

**Table S3.** Top five recommended combinations of Maxent model parameter settings and their associated performance metrics for the northern whitebrush (*Aloysia gratissima*)  baseline species distribution model determined using the ‘ENMeval’ package in R v 4.1.2. (Muscarella et al., 2014; R Core Team, 2022).

| Feature class^a^ | Regularization | Avg test AUC^b^ | Test – train AUC | Continuous Boyce Index | Δ AIC_c_^c^ |
| --- | --- | --- | --- | --- | --- |
| LQHT | 1.5 | 0.74 | 0.04 | 0.81 | 0 |
| LQH | 1.5 | 0.74 | 0.03 | 0.80 | 7.36 |
| LQH | 2 | 0.73 | 0.03 | 0.80 | 7.76 |
| LQHT | 2 | 0.74 | 0.04 | 0.82 | 9.90 |
| LQ | 0.25 | 0.72 | 0.04 | 0.76 | 16.22 |

^a^Feature types estimated in Maxent are: linear (L), quadratic (Q), hinge (H), and threshold (T).

^b^Area Under the Curve.

^c^Akaike Information Criterion adjusted for small sample sizes.

**Table S4.** Top five recommended combinations of Maxent model parameter settings and their associated performance metrics for the southern whitebrush (*Aloysia gratissima*)  baseline SDM determined using the ‘ENMeval’ package in R v 4.1.2 (Muscarella et al., 2014; R Core Team, 2022).

| Feature class^a^ | Regularization | Test AUC^b^ | Test – train AUC | Continuous Boyce Index | Δ AIC_c_^c^ |
| --- | --- | --- | --- | --- | --- |
| LQHT | 1.5 | 0.87 | 0.03 | 0.83 | 0 |
| LQH | 1.5 | 0.87 | 0.03 | 0.79 | 3.35 |
| LQH | 1 | 0.87 | 0.03 | 0.77 | 4.91 |
| LQHT | 2 | 0.87 | 0.03 | 0.81 | 7.29 |
| LQH | 2 | 0.87 | 0.03 | 0.78 | 7.82 |

^a^Feature types estimated in Maxent are: linear (L), quadratic (Q), hinge (H), and threshold (T).

^b^Area Under the Curve.

^c^Akaike Information Criterion adjusted for small sample sizes.

**Table S5.** Binary thresholds for conversion of continuous probability of suitability predictions as determined by the maximum test sensitivity plus specificity threshold selection rule for the median of 30 replications for all presented Maxent species distribution models for whitebrush (*Aloysia gratissima*) (Liu et al., 2013).

| Model | North population | South population |
| --- | --- | --- |
| Baseline | 0.4273 | 0.2250 |
| Spatial transfer | 0.4088 | 0.2148 |
| Temporal transfer (2041) | 0.4416 | 0.2633 |
| Temporal transfer (2061) | 0.4082 | 0.2471 |
| Temporal transfer (2081) | 0.3867 | 0.2817 |

**Table S6.** Global climate models from the CMIP version 6 release selected to create an ensemble mean to represent future climate scenarios for temporal MaxEnt model projections for whitebrush (*Aloysia gratissima*). Global climate models were selected based on guidance by Mahoney et al. to incorporate models from both conservative and liberal ends of the spectrum (Fick & Hijmans 2017; Mahoney et al. n.d.).

| Model name | | Developing institution | Citation | |
| --- | --- | --- | --- | --- |
| ACCESS-ESM1-5 | Commonwealth Scientific and Industrial Research Organization (Australia) | | | Ziehn et al. 2020 |
| CNRM-ESM2-1 | Centre National de Recherches Meteorologiques and Centre Europeen de Recherche et de Formation Avancee en Calcul Scientifique (France) | | | Seferian et al. 2019 |
| EC-Earth-Veg-L | EC Earth Consortium (USA) | | | Doscher et al. 2022 |
| GFDL-ESM4 | National Oceanic and Atmospheric Administration, Geophysical Fluid Dynamics Laboratory (USA) | | | Dunne et al. 2020 |
| GISS-E2-1G | Goddard Institute for Space Studies (USA) | | | Kelley et al. 2020 |
| MIROC6 | Japan Agency for Marine-Earth Science and Technology, Atmosphere and Ocean Research Institute, National Institute for Environmental Studies and RIKEN Center for Computational Science (Japan) | | | Tatebe et al. 2018 |
| MIROC-ES2L | European Geosciences Union (Europe) | | | Hajima et al. 2020 |
| MPI-ESMI-2-HR | Max Planck Institute for Meteorology (Germany) | | | Muller et al. 2018 |
| MRI-ESM2-0 | Meteorological Research Institute (Japan) | | | Yukimoto et al. 2019 |

**Table S7.** Test statistics resulting from the Kolmogorov-Smirnov two sample analysis of the probability of suitability response curves for whitebrush (Aloysia gratissima) generated from our baseline MaxEnt models built with 30 replications (Berger & Zhou, 2014).

| Bioclimatic variable | Schoener’sD statistic | P statistic |
| --- | --- | --- |
| 0 | 0.22677 | <0.001 |
| 2 | 0.17582 | <0.001 |
| 3 | 0.27622 | <0.001 |
| 9 | 0.18831 | <0.001 |
| 13 | 0.28721 | <0.001 |
| 14 | 0.26923 | <0.001 |
| 18 | 0.37612 | <0.001 |

**Table S8**. Variable contribution to principal component axis explained variation extracted from the principal component analysis on climatic conditions at known locations of whitebrush (*Aloysia gratissima*). Analysis included the northern and southern population occurrences and variables included in final species distribution models.

| Biovariable  ID | PC 1  (%) | PC 2  (%) |
| --- | --- | --- |
| 2 | 16.99 | 10.97 |
| 3 | 1.02 | 46.54 |
| 9 | 5.09 | 27.37 |
| 13 | 19.81 | 4.62 |
| 14 | 19.02 | 9.77 |
| 18 | 17.42 | 0.26 |
| 0 | 20.65 | 0.46 |

**Figure S1.** Variable contribution to baseline whitebrush (*Aloysia gratissima*) population models characterizing the ecological niche of the northern distribution (blue) and the southern distribution (yellow). Percent contribution to MaxEnt model performance was determined by a jackknife process over 30 replications.


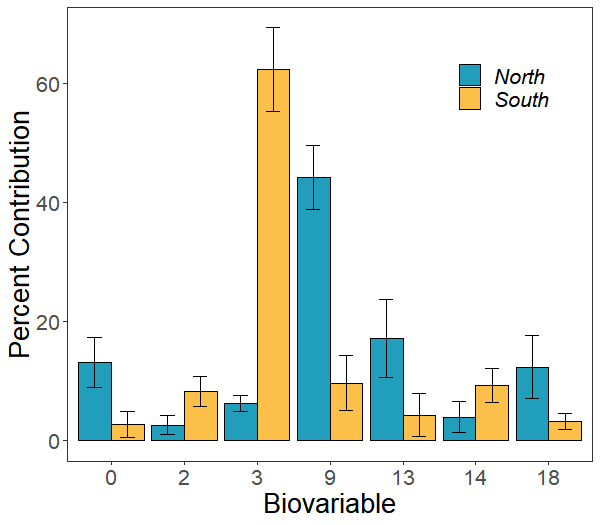


**Figure S2.** Continuous dissimilarity rating of climate conditions displayed as quantiles in 20% intervals in the (a) northern and (b) southern distributions of whitebrush (*Aloysia gratissima*).


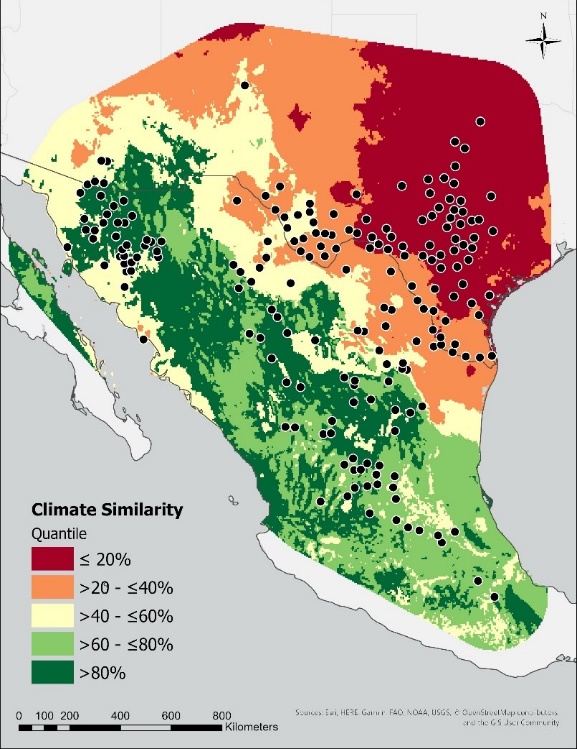

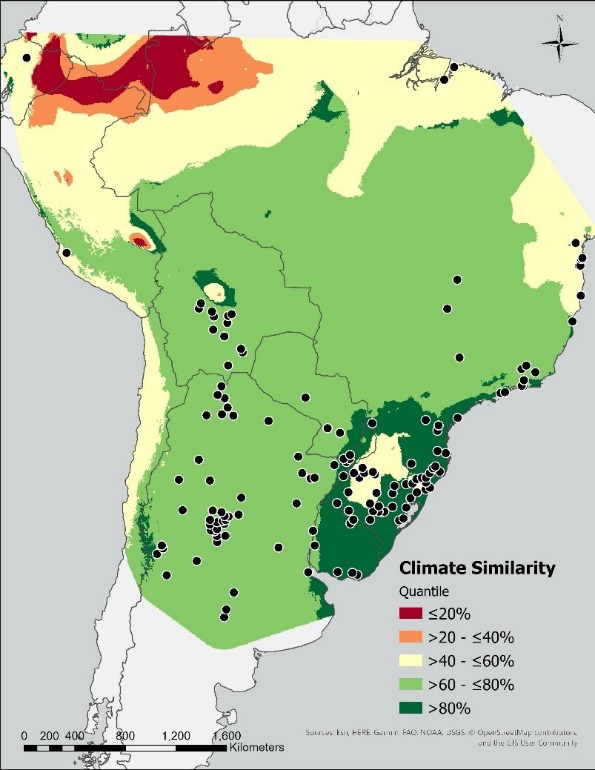


**b**

**a**

**Figure S3.** Principal component analysis (PCA) conducted on climate conditions represented by the bioclimatic predictor variables (Fick & Hijmans 2017) included in our final baseline MaxEnt models extracted at occurrence locations in the northern (blue) and southern (yellow) whitebrush (*Aloysia gratissima*) distributions. Principal components (PC) and the percentage of variation explained by each PC are presented on the X and Y axes.


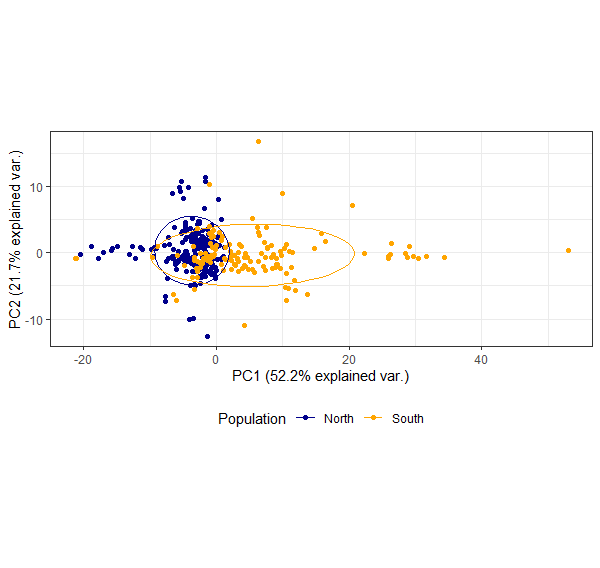

Supplement: Supplementary file 1 — Appendix S1: ece372816‐sup‐0001‐AppendixS1.docx. [file ECE3-16-e72816-s001.docx]
